# Cognitive Improvement and Safety Assessment of a Dietary Supplement Containing Propolis Extract in Elderly Japanese: A Placebo-Controlled, Randomized, Parallel-Group, Double-Blind Human Clinical Study

**DOI:** 10.1155/2021/6664217

**Published:** 2021-02-24

**Authors:** Takashi Asama, Toshihito Hiraoka, Akio Ohkuma, Nobuaki Okumura, Ayanori Yamaki, Katsuya Urakami

**Affiliations:** ^1^Institute for Bee Products and Health Science, R&D Department, Yamada Bee Company, Inc., Okayama, Japan; ^2^Research Center for Immunological Analysis, Inc., Okayama, Japan; ^3^Department of Biological Regulation, School of Health Science, Faculty of Medicine, Tottori University, Tottori, Japan

## Abstract

*Objectives*. This study aimed to evaluate the effect of propolis on cognitive function in elderly Japanese with a placebo-controlled design. *Material and Methods*. This study was performed on 79 elderly Japanese. Participants orally received either a placebo or dietary supplement containing propolis extract for 24 weeks. Cognitive function assessed by Cognitrax and various blood or urine markers were measured at pre- and postadministration. *Results and Conclusion*. Eligible data from 68 subjects (placebo: 33, propolis: 35) who completed the study were analyzed. Compared to the placebo group, the propolis group showed significant improvement in verbal memory in Cognitrax (*P*=0.028). Total cholesterol, LDL cholesterol, urea nitrogen, creatinine, and uric acid were significantly improved in the propolis group compared to the placebo group (*P* = 0.011, *P* = 0.004, *P* = 0.048, *P* = 0.045, and *P* = 0.005, respectively). However, urea nitrogen, creatinine, and uric acid fluctuated within the normal level. Furthermore, a subgroup analysis was performed on those with higher than 100 of the standardized score of the neurocognitive index indicated by the overall Cognitrax score. Significant improvements in the propolis group compared to placebo were confirmed in verbal memory (*P* = 0.007) and processing speed as indications for information processing ability, complex attention, and concentration (*P* = 0.029). No side effects were observed in any of the groups. This study demonstrates that propolis is effective in improving cognitive functions such as memory, information processing, complex attention, and concentration in elderly Japanese.

## 1. Introduction

In Japan, where the population is aging, the number of patients with dementia continues to increase. The number of dementia patients is estimated at about 4.4 million as of 2010, and the number of mild cognitive impairment (MCI), which is a predementia, will be about 3.8 million [1]. Annual economic loss by dementia is estimated as approximately 14.5 trillion yen in 2014 and to be about 24 trillion yen by 2060 in Japan [2]. Furthermore, the number of dementia patients in Japan is estimated at about 6.5 to 7 million in 2025 and about 8 to 9 million in 2040, which is a major social problem [3].

Dementia is caused by chronic and progressive brain disease associated with a syndrome consisting of many higher brain dysfunctions such as memory, thinking, disorientation, understanding, calculation, learning, language, and judgment [4]. The only therapeutic method is a drug that temporarily delays the progression, and there is no fundamental therapeutic method. Therefore, prevention at a stage where age-related cognitive decline is mild and at a reversible level is very important. In many cases of dementia, the main cause has not been completely clarified yet, but age-related cognitive decline involves multiple factors, including inflammation [5, 6], oxidative stress [7], neurotransmitter decline [8], and beta-amyloid accumulation [9]. Therefore, improving these multiple factors is considered important for maintaining and improving cognitive function.

Propolis is a substance that honeybees make from plant resin as the main raw material for maintaining the hygienic environment of their nests. Propolis used in this study has been reported to have antioxidant [10] and anti-inflammatory effects [11] in humans. In addition, an inhibitory effect on beta-amyloid accumulation *in vivo* [12] and an increasing effect on the brain-derived neurotrophic factor (BDNF) *in vitro* have been reported [13]. Regarding cognitive function, propolis has been reported to improve cognitive function in the elderly living in the Tibetan Plateau, China, which is prone to the development of dementia due to a hypoxic environment [11]. However, the effect of propolis on cognitive function has not been verified at the normal altitude where the Japanese live. Therefore, we examined the effect of propolis on the cognitive function of elderly Japanese in a placebo-controlled, randomized, parallel-group, double-blind human clinical study.

## 2. Material and Methods

### 2.1. Subjects

K.S.O. Co., Ltd. (Tokyo, Japan) recruited the subjects. The main inclusion and exclusion criteria are presented as follows:

  Inclusion criteria:
(1) Men and women between the ages of 60 and 79 at the time of obtaining their consent
(2) Mini-Mental State Examination (MMSE) with 24 to 29 points
(3) Individuals who are aware of living with forgetfulness or have been pointed out by others
  Exclusion criteria:
(1) Individuals who have been diagnosed with dementia by a medical doctor or who have a disease that may affect cognitive function
(2) Individuals who are being currently treated for dementia or in the past
(3) Individuals who have regularly taken drugs that may affect cognitive function (such as first-generation antihistamines, benzodiazepines, sedatives, opiates, stabilizers, antidepressants, cholinergic drugs, anticholinergic drugs, and prescription anti-inflammatory drugs) and any other influential agents
(4) Individuals who are considered inappropriate as subjects as a result of the MRI examination
(5) Individuals who have a current or previous history of mental disorders (including depressive symptoms) or cerebrovascular diseases
(6) Individuals taking warfarin
(7) Individuals who regularly use dietary supplements and health foods (including foods with functional claims) that may affect cognitive function
(8) Based on the subject background questionnaire, those with an extremely irregular lifestyle, such as eating and sleeping
(9) Individuals with a geriatric depression scale (Short Version-Japanese, GDS-SJ) score of 6 or higher points
(10) Individuals who have a current or previous history of alcohol dependence
(11) Individuals with high daily alcohol addiction (more than 14 bottles of 350 mL of beer or 180 mL of wine per week)
(12) Individuals with a current or history of serious illness, such as diabetes, liver disease, kidney disease, heart disease, and more
(13) Individuals with a current or history of drug addiction or food allergy
(14) Individuals with color blindness and hearing impairment even at close range
(15) Individuals with functional problems in both hands

### 2.2. Test Food

A dietary supplement containing propolis extract (artepillin C, 57.68 mg; culifolin, 0.95 mg) as standard components in a daily dose of 6 soft capsules was prepared. Placebo was made by replacing propolis with starch. The propolis and placebo capsules had an identical appearance so that they are indistinguishable. The nutritional content of both preparations is shown in Table 1.

### 2.3. Study Design

The present study was reviewed and approved by the Ethics Committee of the Nihonbashi Cardiology Clinic on March 11, 2019 (approval no. NJI-019-03-01). It was conducted in accordance with the Declaration of Helsinki (revised at the 64^th^ WMA General Assembly, Fortaleza, Brazil, October 2013) and Ethical Guidelines for Medical and Health Research Involving Human Subjects (Ministry of Health, Labor and Welfare, 2017). The study protocol was registered on the University Hospital Medical Information Network (UMIN 000036177).

The study design was a placebo-controlled, randomized, parallel-group, double-blind comparative human clinical study. The objectives and methods of the study were fully explained to the subjects, and written consent was obtained. Seventy-nine subjects, who met the inclusion criteria and did not fall under any of the exclusion criteria were randomly assigned to two groups and distributed equally based upon their ages, gender, body mass index (BMI), and MMSE. Since this was the first study to investigate the effect of propolis intake on cognitive function using Cognitrax, it was not possible to calculate the sample size. However, we referred to other clinical studies which evaluated cognitive function to estimate the number of subjects. [14, 15]. The Allocation Manager created a correspondence table in which the subject ID was assigned to groups A and B that was sent to the Test Food Assignment Manager who was not directly involved in the study. The Test Food Allocation Manager prepared a matching table with the test food codes and the subject ID based on the correspondence table obtained from the Allocation Manager and then sent it to the study-conducting site. The Test Food Allocation Manager retained the original table (correspondence table with subject ID-group-test food code) strictly confidential without disclosing it to anyone until the data was fixed.

Preadministration test (height, weight, blood pressure, pulse, MMSE by clinical psychologist, MRI, GDS-SJ, Cognitrax, and various blood and urine tests) was conducted in March–May 2019, and 6 placebo or propolis capsules were ingested daily with water for 24 weeks from June to November 2019. Furthermore, after 24 weeks of administration, height, weight, blood pressure, pulse, Cognitrax, and various blood and urine markers were measured again. In addition, during the test period, a daily log was kept in order to record the test food intake, presence or absence of changes in the body condition, lifestyle routines such as sleep and exercise status, intake of the drug and/or health foods, and the state of interpersonal exchange.

### 2.4. Evaluation of Cognitive Function Using Cognitrax

Cognitrax is a general cognitive test based on CNS vital signs [16]. In the present study, the verbal memory test (VBM), the visual memory test (VIM), the finger tapping test (FTT), the symbol digit coding test (SDC), the Stroop test (ST), the shifting attention test (SAT), the continuous performance test (CPT), and the four-part continuous performance test (FPCPT) of the Cognitrax system were carried out. The test results were evaluated with the standardized score (average of 100) converted compared to the same age. Based on these tests, the neurocognitive index, composite memory, verbal memory, visual memory, reaction time, complex attention, sustained attention, simple attention, cognitive flexibility, psychomotor speed, processing speed, motor speed, executive function, and working memory were calculated. The neurocognitive index is composed of composite memory, psychomotor speed, reaction time, complex attention, and cognitive flexibility, while composite memory is composed of verbal memory and visual memory.

### 2.5. Blood Test

#### 2.5.1. Blood Biochemistry

White blood cell count, red blood cell count, hemoglobin, hematocrit, platelet count, total protein, albumin, total bilirubin, AST, ALT, LDH, *γ*-GTP, ALP, urea nitrogen, uric acid, creatinine, total cholesterol, HDL-Cholesterol, LDL cholesterol, triglyceride, sodium, potassium, chloride, HbA1c, and fasting blood glucose were measured. White blood cells, red blood cells, hemoglobin, hematocrit, and platelets were measured using Sysmex XE-2100 automated hematology analyzer (Sysmex, Kobe, Japan). Total protein, albumin, total bilirubin, AST, ALT, LDH, *γ*-GTP, ALP, urea nitrogen, uric acid, creatinine, total cholesterol, HDL-cholesterol, LDL cholesterol, triglyceride, sodium, potassium, and chloride were measured with LABOSPECT 008*α* (Hitachi High-Tech, Tokyo, Japan) and JCA-BM8060 (JEOL Ltd., Tokyo, Japan). HbA1c and fasting blood glucose levels were measured with JCA-BM9030 and JCA-BM9130 (JEOL Ltd., Tokyo, Japan).

#### 2.5.2. Specific Measurement

In addition, as inflammation and mercury toxicity are associated with cognitive function [5] and Alzheimer's disease [17], serum TNF-*α* and mercury blood levels were analyzed. TNF-*α* was measured by MESO Quick Plex SQ 120 and V-PLEX Proinflammatory Panel 1 human Kit (Meso Scale Discovery, Maryland, United States). Mercury was measured by a Mercury Analyzer MA-3000 model (Nippon Instruments, Tokyo, Japan).

### 2.6. Urine Analysis

Urinary protein, glucose, occult blood reaction, urobilinogen, bilirubin, ketone bodies, pH, and specific gravity were measured with US-3500MS (Eiken Chemical, Tokyo, Japan).

### 2.7. Statistical Analysis

All values indicate mean ± standard deviation. The intragroup comparison between pre- and postadministration for 24 weeks was assessed by Wilcoxon signed-rank test. The propolis and placebo groups were compared with the Wilcoxon rank-sum test. Changes (Δ) between pre- and postadministration were compared between groups in a similar way. All statistical calculations were based on the bidirectional analysis. SPSS Ver.25 (IBM) was used for all statistical analyses.

## 3. Results

### 3.1. Subjects

A total of 79 subjects (40 in the placebo group and 39 in the propolis group) participated in the study, with 1 in the placebo group and 2 in the propolis group dropped out for personal reasons. In addition, 8 subjects were excluded from analysis due to the following reasons:

1. Liver markers such as AST and ALT are related to cognitive function [18]. Two subjects (1 in placebo, 1 in propolis) were excluded due to an outlier (>mean + 3SD) of liver disorder marker changes (Δ) between preadministration and postadministration.
2. Blood pressure is also related to cognitive function [19]. One subject in the placebo group was excluded due to an outlier (<mean-3SD) of blood pressure changes (Δ) between preadministration and postadministration.
3. Mercury related to Alzheimer's disease [17] was an outlier (<mean-3SD) at changes (Δ) between preadministration and postadministration in 1 subject (placebo).
4. The body weight change of 1 subject in the placebo group was an outlier (>mean + 3SD) between preadministration and postadministration.
5. The change in fasting blood glucose in 1 subject in the placebo group was an outlier (>mean + 3SD) between preadministration and postadministration.
6. One subject (propolis group) unreasonably reduced lunch intake due to weight gain. It is very likely that the lifestyle of the 7 subjects above will not be constantly maintained during the study period. Judging by the accuracy of checking the effects on cognitive function, it can be negatively affected. Hence, we excluded them from the analysis.
7. In the majority (8 items) of the 14 main items of Cognitrax, 1 subject (placebo group) whose change each score of Cognitrax before and after ingestion was an outlier (>mean + 3SD). The reason may be that this subject did not understand the contents of Cognitrax before ingestion or was not motivated for the test.

**Figure 1:**
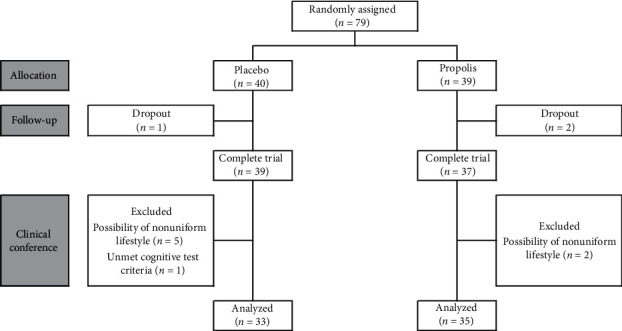
Trial flow diagram.

Overall, the total number of subjects in the analysis was 68.  Figure 1 describes the trial flow diagram, and Table 2 shows the background of the subjects. There were no significant differences between groups in terms of age, gender, BMI, and MMSE.

### 3.2. Evaluation of Cognitive Function (Cognitrax)

The results are shown in Table 3. A significant improvement was observed in the change (Δ) of verbal memory in the propolis group compared to the placebo group (*P*=0.028). No significant difference was observed in other items between the two groups.

### 3.3. Blood Test

The results are shown in Table 4. The changes (Δ) in total cholesterol, LDL cholesterol, urea nitrogen, creatinine, and uric acid indicate significant changes in the propolis group compared to the placebo group (*P* = 0.011, *P* = 0.004, *P* = 0.048, *P* = 0.045, and *P* = 0.005, respectively). However, urea nitrogen, creatinine, and uric acid fluctuated within a normal level. No significant differences of other parameters between the placebo and propolis group were observed (data not shown regarding some blood and urine tests).

### 3.4. Subgroup Analysis

The accuracy of MCI discrimination with Cognitrax is not high at approximately 74% [20], and the sensitivity to detect changes in cognitive function in subjects with impaired cognitive function may not be high. In other words, subjects with high cognitive function are considered to be more likely to confirm the effectiveness of propolis intake than those with impaired cognitive function. Therefore, a subgroup analysis was performed on those who had a standardized score of the neurocognitive index, which is the overall score of Cognitrax, higher than 100, that is, those with higher than 100 of the standardized score of the neurocognitive index indicated by the overall Cognitrax score. These subjects had higher cognitive function than the average of the same age group. There were no significant differences between the groups in terms of age, gender, BMI, and MMSE (Table 5). As indicated in Table 6, significant improvements in the change (Δ) of the propolis group compared to placebo were detected in verbal memory (*P* = 0.007) and processing speed (*P* = 0.029). No statistically significant differences were observed for other parameters between the placebo and propolis group (data not shown).

### 3.5. Safety

Although 53 adverse events were noted in this study, the responsible physician determined that none of them had a causal relationship with the food study. In addition, as the results of the blood and urine tests were within the range of physiological fluctuations, it was concluded that there were no safety concerns.

## 4. Discussion

As a result of evaluating the effect of propolis intake on cognitive function, a significant improvement in verbal memory was found in the propolis group compared to the placebo group. The present study is the first study to confirm the beneficial effect of propolis on cognitive function at the normal altitude in a short period of intake time. In addition, propolis intake had impacts on total cholesterol, LDL cholesterol, urea nitrogen, creatinine, and uric acid levels. However, urea nitrogen, creatinine, and uric acid fluctuated within a normal level.

Zhu et al. reported improved cognitive function and the inflammatory marker by propolis supplementation for two years in the elderly living in the Tibetan Plateau, China [11]. This report supports the results of this study. The onset of dementia has been reported to be a general decline in cognitive function after a decline in memory, such as episodic memory and semantic memory (including verbal memory) [21]. In addition, Welsh et al. performed various cognitive function tests in three groups of healthy people, mild Alzheimer's disease (AD), and moderate AD patients to predict disease progression [22]. As a result, delayed memory is reported to be particularly sensitive as an index for early identification of AD [22]. From the above, it is considered that improving verbal memory, especially delayed memory, is very important in preventing dementia. Cognitrax verbal memory task score is calculated from the number of correct answers of immediate memory and delayed memory, which is a memory for a few minutes. While statistically significant data are not shown here, there is tendency to improve correct answers in the immediate and delayed memory. Additional studies will be needed to confirm if propolis acts in a well-balanced manner to improve both immediate and delayed memory. On the other hand, there is a difference in TNF-*α* level in the present study and Zhu et al. [11]. We think two possible reasons for this difference are as the following:

1. The present study was conducted at the normal altitude, not in high areas where inflammation is more likely promoted.
2. The study of Zhu et al. [11] took place over a period of two years. The intake period in this study was 24 weeks, and much shorter than that of Zhu et al.

If the study would be carried out under the condition that the inflammatory state is promoted and the test period is long, the anti-inflammatory effect of propolis may be clearly detected.

Possible mechanisms to improve the cognitive function of propolis are not only anti-inflammatory effect but also neuroprotective action from toxicity caused by beta-amyloid or oxidative stress and promoting the effect of brain-derived neurotrophic factor (BDNF) production. Unpublished data have shown that propolis has an inhibitory effect on amyloid beta-1-42 aggregation and has been confirmed to have an inhibitory effect on amyloid beta 1–42 neurotoxicity in the PC-12 cell line. It is considered to help improve cognitive function by alleviating neurotoxicity caused by amyloid beta. In addition, propolis has a neuroprotective effect from excess free radicals (ROS) [13] and an antioxidant effect in model mice with cerebral ischemia [23]. Therefore, propolis may suppress cognitive decline by protecting nerves through antioxidant activity. Regarding BDNF, high BDNF expression has been reported to slow the rate of cognitive decline [24] and propolis promotes its production in SH-SY5Y cells [13]. From these findings, it is possible that propolis contributes to the improvement of cognitive function by promoting the production of BDNF.

In addition, likely to this study, the improvement of cholesterol level was observed by administering propolis to rats, which supports and is consistent with the results of this study [24]. In the same paper, the inhibitory effect of hydroxymethylglutaryl-CoA (HMG-CoA) reductase, a cholesterol synthase, is also reported [25]. Therefore, the inhibitory effect of HMG-CoA reductase is considered to be one of the mechanisms of cholesterol reduction by propolis. In addition, high LDL cholesterol levels increase the risk of AD shown in a meta-analysis [26]. Elevated levels of LDL-C and TC cause the extracellular deposition of amyloid beta, hindering neuronal synaptic connections in the brain and increasing the risk of AD [27]. High LDL cholesterol and low HDL cholesterol levels have been associated with higher cerebral amyloid beta levels [28]. These studies suggest that the cholesterol-lowering effect of propolis may also contribute to improving cognitive function.

According to a subgroup analysis of the Cognitrax in the group with those with higher than 100 of the standardized score of the neurocognitive index indicated by the overall Cognitrax score, significant improvements were also found in verbal memory. This was consistent with the primary analysis in addition to improving processing speed (the ability to process information quickly). The *P* values between the two groups in the scores of immediate and delayed memory tasks that constitute verbal memory were 0.544 and 0.010, respectively (data not shown). This suggests propolis intake may be more effective in improving delayed memory than an immediate one. The processing speed for which an improvement was observed in addition to the verbal memory is calculated by “correct SDC answer-incorrect SDC answer.” The SDC displays 8 corresponding symbols and numbers at the top of the screen, and only 8 symbols at the bottom. Subjects enter numbers corresponding to the symbols at the bottom and are evaluated for their ability to process information, complex attention, and concentration. Therefore, improving processing speed suggests improving processing capacity, complex attention, and concentration. Specific examples of information processing in everyday life include “fast turning the steering wheel when it is dangerous” and “fast and accurate detection of danger and risk while driving.” In terms of attention, “safe driving for a long time” and “without overlooking a red light” are mentioned.

This study has several limitations that should be noted: (1) the lower effective dose and dose dependence are not clear and (2) the detailed mechanism and associated component for improving cognitive function are not characterized. Further studies are needed to clarify these aspects.

## 5. Conclusion

Propolis intake improves not only verbal memory but also information processing, attention, and concentration in a group with high cognitive function. In addition, no side effects were shown by propolis ingestion. Thus, propolis is considered a very safe food.

## Figures and Tables

**Table 1:** Nutritional content.

	Placebo	Propolis
Energy (kcal)	6.48	7.74
Proteins (g)	0.12	0.36
Lipids (g)	0.06	0.30
Carbohydrates (g)	1.86	1.44
Salt equivalent (mg)	1.14	1.08

**Table 2:** Baseline characteristics of the study population.

	Placebo	Propolis	*P* value
	(*n* = 33)	(*n* = 35)	
Age	66.1 ± 4.5	66.6 ± 3.9	0.614
Gender (male/female)	18/15	16/19	—
BMI (kg/m^2^)	22.8 ± 3.7	22.6 ± 2.7	0.917
MMSE	27.5 ± 1.4	27.5 ± 1.3	0.980

Values are mean ± SD. BMI, body mass index; MMSE, Mini-Mental State Examination.

**Table 3:** Cognitrax.

		Placebo	Propolis
		(*n* = 33)	(*n* = 35)
*Neurocognitive index*	Baseline	102.3 ± 6.7	100.2 ± 7.5
	Week 24	106.3 ± 5.8 ^##^	104.8 ± 6.8 ^##^
	Δ	4.0 ± 5.4	4.6 ± 5.4

*Composite memory*	Baseline	98.1 ± 12.6	96.6 ± 10.1
	Week 24	104.6 ± 17.0 ^#^	107.9 ± 13.6 ^##^
	Δ	6.5 ± 14.8	11.3 ± 10.4

*Verbal memory*	Baseline	94.5 ± 15.7	95.1 ± 11.9
	Week 24	103.5 ± 19.9 ^##^	111.2 ± 12.6 ^##^
	Δ	9.0 ± 13.5	**16.1** **±** **10.7**^∗^

*Visual memory*	Baseline	102.8 ± 11.3	99.7 ± 11.9
	Week 24	105.1 ± 16.0	102.5 ± 14.0
	Δ	2.3 ± 15.7	2.9 ± 14.6

*Reaction time*	Baseline	99.8 ± 7.7	95.1 ± 9.2
	Week 24	102.8 ± 10.9	96.7 ± 9.6^∗^
	Δ	3.0 ± 9.7	1.6 ± 8.4

*Complex attention*	Baseline	107.0 ± 7.9	106.9 ± 11.1
	Week 24	109.9 ± 9.5 ^#^	108.4 ± 8.3
	Δ	2.9 ± 12.9	1.5 ± 9.8

*Sustained attention*	Baseline	108.7 ± 11.8	106.3 ± 12.2
	Week 24	108.3 ± 19.2	108.8 ± 9.4
	Δ	−0.4 ± 15.7	2.5 ± 8.9

*Simple attention*	Baseline	103.9 ± 12.0	105.9 ± 6.3
	Week 24	107.2 ± 4.0	106.9 ± 4.2
	Δ	3.3 ± 12.4	0.9 ± 7.0

*Cognitive flexibility*	Baseline	101.8 ± 7.5	100.5 ± 11.8
	Week 24	105.5 ± 7.5 ^#^	103.0 ± 9.8
	Δ	3.7 ± 9.2	2.5 ± 8.8

*Psychomotor speed*	Baseline	105.0 ± 12.2	102.4 ± 11.0
	Week 24	108.6 ± 11.4 ^#^	107.3 ± 10.8 ^##^
	Δ	3.6 ± 7.5	4.9 ± 6.0

*Processing speed*	Baseline	112.9 ± 11.1	112.1 ± 8.8
	Week 24	113.2 ± 9.9	114.0 ± 9.1
	Δ	0.3 ± 9.4	1.9 ± 7.7

*Motor speed*	Baseline	98.2 ± 11.8	95.4 ± 12.6
	Week 24	102.3 ± 12.1 ^##^	100.3 ± 12.3 ^##^
	Δ	4.2 ± 7.5	4.9 ± 6.6

*Executive function*	Baseline	101.7 ± 7.9	100.1 ± 12.4
	Week 24	105.6 ± 6.3 ^#^	102.8 ± 9.9
	Δ	3.9 ± 8.5	2.7 ± 8.9

*Working memory*	Baseline	104.4 ± 15.9	103.4 ± 14.0
	Week 24	106.1 ± 15.0	105.0 ± 9.6
	Δ	1.8 ± 17.5	1.6 ± 10.1

Values are mean ± SD.  ^#^*P* < 0.05 compared to placebo.  ^#^*P* < 0.05 and  ^##^*P* < 0.01 compared to baseline. Δ, change compared to baseline.

**Table 4:** Blood test.

		Placebo	Propolis
		(*n* = 33)	(*n* = 35)
*Total cholesterol (mg/dL)*	Baseline	209.5 ± 39.8	227.2 ± 23.8^∗^
	Week 24	229.1 ± 37.9 ^##^	235.8 ± 23.0 ^#^
	Δ	19.6 ± 24.2	**8.7** **±** **18.5**^∗^

*LDL cholesterol (mg/dL)*	Baseline	123.7 ± 28.5	137.3 ± 21.9^∗^
	Week 24	141.1 ± 31.7 ^##^	143.0 ± 21.0 ^#^
	Δ	17.4 ± 19.8	**5.7** **±** **14.2**^∗^^∗^

*HDL cholesterol (mg/dL)*	Baseline	64.2 ± 22.1	67.7 ± 16.5
	Week 24	67.1 ± 21.0 ^#^	69.1 ± 16.2
	Δ	2.8 ± 8.1	1.5 ± 6.3

*Urea nitrogen (mg/dL)*	Baseline	14.7 ± 2.7	15.4 ± 3.6
	Week 24	15.2 ± 3.0	14.5 ± 3.0
	Δ	0.5 ± 2.6	**-0.9** **±** **2.8**^∗^

*Creatinine (mg/mL)*	Baseline	0.788 ± 0.182	0.764 ± 0.160
	Week 24	0.834 ± 0.187 ^##^	0.779 ± 0.167
	Δ	0.046 ± 0.065	**0.015** **±** **0.061**^∗^

*Uric acid (mg/mL)*	Baseline	5.0 ± 1.2	5.0 ± 1.0
	Week 24	5.2 ± 1.3 ^#^	4.8 ± 1.1
	Δ	0.3 ± 0.6	**-0.2** **±** **0.6**^∗^^∗^

*TNF-α (pg/mL)*	Baseline	1.7 ± 0.3	1.7 ± 0.5
	Week 24	2.7 ± 0.6 ^##^	2.5 ± 0.6 ^##^
	Δ	1.0 ± 0.5	0.8 ± 0.5

*Mercury (μg/dL)*	Baseline	0.86 ± 0.54	0.93 ± 0.63
	Week 24	0.65 ± 0.53 ^##^	0.73 ± 0.56 ^##^
	Δ	−0.22 ± 0.22	−0.21 ± 0.39

Values are mean ± SD.  ^*∗∗*^*P* < 0.01 compared to placebo.  ^#^*P* < 0.05 and  ^##^*P* < 0.01 compared to baseline. Δ, change compared to baseline.

**Table 5:** Baseline characteristics of the study population (subgroup analysis).

	Placebo	Propolis	*P* value
	(*n* = 18)	(*n* = 16)	
Age	66.2 ± 4.6	66.7 ± 4.6	0.742
Sex (male/female)	10/8	7/9	―
BMI (kg/m^2^)	23.0 ± 2.9	22.5 ± 2.6	0.717
MMSE	27.7 ± 1.4	27.7 ± 1.1	0.708

Values are mean ± SD. BMI, body mass index; MMSE, Mini-Mental State Examination.

**Table 6:** Cognitrax (subgroup analysis).

		Placebo	Propolis
		(*n* = 18)	(*n* = 16)
*Verbal memory*	Baseline	102.4 ± 9.3	97.8 ± 11.2
	Week 24	108.9 ± 13.8	115.9 ± 7.3
	Δ	6.5 ± 13.6	**18.1** **±** **7.0**^∗^^∗^

*Processing speed*	Baseline	118.2 ± 9.5	113.9 ± 8.5
	Week 24	115.3 ± 10.5	117.8 ± 7.5
	Δ	−2.9 ± 10.2	**3.8** **±** **6.5**^∗^

Values are mean ± SD.  ^*∗*^*P* < 0.05,  ^*∗∗*^*P* < 0.01 compared to placebo. Δ, change compared to baseline.

## Data Availability

The data used to support the findings of this study have been deposited in the private folder of Yamada Bee Company, Inc.
